# Historicizing Indian psychiatry

**DOI:** 10.4103/0019-5545.55963

**Published:** 2005

**Authors:** Amit Ranjan Basu

**Affiliations:** MBBS, Doctoral Candidate Centre for Historical Studies, Jawaharlal Nehru University, New Delhi

**Keywords:** History of Indian psychiatry, colonial and post-colonial psychiatry

## Abstract

Our historical endeavour to map Indian psychiatry has largely remained linear, positivistic and evolutionary. Whether it starts from the ancient times or modern, it shows our past as a tale of victory for the western science, without questioning the borrowed paradigm. The use of historical methods for serious enquiry of psychiatry has been ignored. Emergence of a new genre of historicism that is critical of both colonialism and psychiatry as a universal science, has raised hopes to critically review the emergence of psychiatric knowledge.

## INTRODUCTION

The history of psychiatry in India has been interpreted in only one way. The development of modern psychiatry has always been seen, especially in colonial and post-colonial times, through the prism of a western science which makes evolutionary and linear progress and where we are perpetually located in a situation of *lack.* This reductionism and positivistic way of interpreting a past needs to be questioned.

To explore *how* the past of Indian psychiatry is represented, the interdisciplinary method of social science is required and historical texts selected from colonial and post-colonial times need to be gleaned. In the recent critique of history of science (particularly in the colonies where ‘sciences’ arrived as a tool for expansion of the empire and to govern the colonized) researchers have raised crucial questions on the omnipotence of the colonial science.^1^–^4^ Analytical tools adopted in these studies challenge the ‘reality’ claimed in the historical narratives of the colonial rulers and expose the underlying assumptions.

## HISTORICISM AND FOUCAULT'S WORK

It is important to say something on historicism first and the rupture brought in the history of psychiatry by Michel Foucault's work.^5^ This would help us to understand better the emergence of current scholarship.

Historicism is a critical movement that insists on the prime importance of historical context to the interpretation of *texts of all kinds* [emphasis mine]. It has developed as a reaction to the practice of deducing truth from first principles about how people must organize themselves socially and politically. Natural laws governing human behaviour at all times are formulated, and cultures evaluated by the degree to which they are appropriate to this ideal pattern. Historicists oppose this tradition which, primarily associated with the Enlighten-ment, stretches in different versions, from the seventeenth century natural-law theorists to the sophistications of Kant and Hegel. Historicists maintain that human nature is too variable for such legislation to be universally applicable. Historicists work to evolve a model for apprehending cultural and social diversity that is different from the scientific, law-governed paradigm of the Enlightenment.^6^ In the sense, this new historical awareness means acknowledging the autonomy of a past in the present.

From the South Asian perspective, social scientist Dipesh Chakrabarty has dealt with the issue of historicism critically in the context of theory. Here Chakrabarty proposes a different dimension to historicism influenced by Foucault. He wrote:

Scholars contemplating the subjects called ‘Indian history’ have often relieved, as it were, the old passions of the ‘struggles of the Enlightenment with superstition’ that Hegel writes about in his *Phenomenology.* They have assumed that for India to function as a nation based on the institutions of science, democracy, citizenship, and social justice, ‘reason’ had to prevail over all that was ‘irrational’ and ‘superstitious’ among its citizens. Historicism has been a very close ally of such thought… How would history, the rational–secular discipline, understand and represent such practices?… But sympathetic or not, these accounts all foreground a separation—a subject–object distinction—between the academic observer–subject and the ‘superstitious’ persons serving as the object of the study.^7^

The history of psychiatry never remained the same after Michel Foucault. All the work done before him can be clubbed as various kinds of evolutionary histories that described asylum practices and its institutional growth, which was to become the discipline of psychiatry later. By seriously questioning these kind of histories, Foucault opened up possibilities for a new range of studies on mental hospitals and psychiatry. Foucault was keen to see how discourses* in a specific context produce *episteme* that govern our thinking in a particular way. Though he generated hostility from mainstream medical historians, Foucault's views were received positively by a section of continental philosophers and social scientists who could see the radicalism with which he related power to a set of discursive practices that depended on the omnipotence of Enlightenment and Reason. It would be useful to look at the context of the emergence of Foucault's major work on madness.

What makes Foucault's work on history of madness so special is his attempt to write the history of both concepts and institutions in a way that blurs the distinction between the two.^9^ Foucault focused on *what goes on* instead of looking at the rationally conceived object of knowledge.

## RECENT INSIGHTS ABOUT OUR PAST

The new historiography that has emerged for the past two decades on science and medicine, particularly on psychiatry and psychoanalysis in India, has examined colonialism in all its complexity. Ashis Nandy^10^ was the first who analysed the psychology of colonialism in India provocatively without following a historical method. He borrowed the interpretative tools of psychoanalysis and ‘focused on the living traditions, emphasizing the dialectic between the classical, the pure and the high-status on the one hand, and the folksy, hybrid and the low-brow on the other’.^10^ His work is no less seminal than Frantz Fanon's^11^—the psychiatrist and revolutionary who focused on the concept of *negritude* and analysed colonial power in Algeria. Nandy^10^ looked at that *form* of colonization, ‘which at least six generations of the Third World have learnt to view as a prerequisite for their liberation. This colonialism *colonizes minds* in addition to bodies and releases forces within colonized societies to alter their cultural priorities once for all’^10^ [emphasis mine].

Gyan Prakash^4^ has offered a radical analysis of colonialism and modernity, which goes beyond the colonial/national binary and explores the issue of how scientific reason worked out a form of governance evoking a new imagination of modern nation-state that mimicked the European model. He said:

Compelled to use universal reason as a particular means of rule, the British positioned modernity in colonial India as an uncanny double, not a copy, of the European original—it was almost the same but not quite. In the colonial context universal claims of science always had to be represented, imposed, and translated into other terms. This was not because Western culture was difficult to reproduce, but because it was dislocated by its functioning as a form of alien power and thus was forced to adopt other guises and languages.^4^

Prakash has innovatively worked out his analysis using Foucauldian tools to bring out how the project of Indian science was, from the very beginning, problematized. He has discussed in detail the issue of translation of science and showed the existence of a critical discourse that was aware of the power differentials in this process. He has argued that in Indian modernity we are negotiating the polarities of secular and religious, community and state, science and culture, whose hybridization have formed the stuff of its historical existence.

In the domain of history of psychiatry in India too, we observe the emergence of this critical historiography. Waltraud Ernst, a psychologist turned social historian, has done major work^12^–^15^ to analyse the issues of early institutionalization, racism, gender and colonial structures. Christiane Hartnack's work has looked into the context of the emergence of psychoanalysis under colonial dominance and the Indian response.^16^ Her critical analysis centred around Girindrasekhar Basu, the first psychoanalyst outside the western world, and other scholars proposing new theories of the Indian psyche.

Girindrasekhar has attracted scholarly attention recently after Nandy's insightful and imaginative analysis.^17^,^18^ While in ‘Savage Freud’ his reading of Girindrasekhar's life delved into an internal critique of Indian psychiatry, the other article has used the symbol of Girindrasekhar's oeuvre as a critique of modern historiography. Kakar^19^ also has observed how Girindrasekhar has modified the Freudian version of psychoanalysis through his innovative practice. Basu^20^ followed up Nandy's analyses on Girindrasekhar and interpreted his Bengali works to bring out his critique of the received science.

Two studies have looked critically into the issue of cannabis use and the colonial power. Mills^21^ has meticulously screened the colonial records of asylums for Indian patients in the nineteenth century, specifically focusing on the cannabis use. His analysis questioned the scientific hypothesis of ‘hemp insanity’ and showed how colonial power influenced this postulation. The other study by Basu^22^ has focused on the questionnaire used by the Indian Hemp Drugs Commission and analysed the evidences given by indigenous medical practitioners and showed how the history of ‘cannabis psychosis’ is constructed.

There is thus a growing corpus of contemporary research on Indian science and medicine that has opened up new possibilities to re-examine the concept of a ‘humanitarian’, ‘universal’ science. These possibilities help us to see our specificity and conceptual growth that make our history not a simple tale of progress, but a complex discourse. Studies in the history of psychiatry have raised doubts about the success of the Enlightenment project and explored conceptual critiques from the historical contexts.

## TEXTS FROM COLONIAL INDIA

In an editorial in the *Indian Medical Gazette*^23^ the medical progress in the nineteenth century was reviewed with a brief passage on ‘Lunatic Asylums’, which said:

It is not, however, till within recent years that we find proper arrangements for the due care of the insane and for the teaching and training of the asylum attendants. We have on previous occasions referred to the changes which are about to take place in the management of the asylums of India, and with the new century we have every reason to expect that a new era is dawning for the insane in India.^23^

Lodge Patch reviewed the nineteenth century Indian psychiatry historically. He followed the development of Punjab Mental Hospital from 1840 to 1930.^24^ His views are typically colonial, where Indians are transformed through custodial care in asylums from their ignorance and superstition. He is astonished that Indians are not interested in the salvation of the mental health of their country, though he did not mention how Indians view mental salvation according to different traditions. He reduced all this into ‘their belief on spirit’! To him it was a humanitarian task to impose the European model for ‘development’.

Berkeley-Hill^25^ in his autobiography wrote a chapter on ‘Ranchi European Mental Hospital’. His narrative described the internal contradictions of colonial administration and posed him as a major protagonist for developing Indian psychiatry on the latest scientific advancements. His history talked of his struggles with the higher colonial authority to bring modern changes. In the epilogue of his book, he wrote:

On the 24th October, 1934, I handed over charge of the Ranchi European Mental Hospital, and like the Cheshire Cat, faded away. I had refused all suggestions of any sort of farewell function. Like H. G. Wells, I feel that leaders should guide as far as they can and then vanish. Looking back on the fifteen years of my leadership I realize only too well that much of my work was slovenly and irritated. But inspite of this, the miserable bear-garden I had taken charge of in October, 1919, had become the *finest mental hospital in Asia,* and a great deal finer than many mental hospitals in Europe^25^ [emphasis mine].

Berkeley-Hill was a founder member of the Indian Psychoanalytical Association and a prolific writer. But his writings strongly supported colonial ideology and he used psychoanalysis as a tool of governance.^26^

Girindrasekhar Bose's article^27^ was written more as a scientific review of the past 25 years' work done in psychology than as a historical narrative. It, however, reflects the contours of a nationalistic science. Colonial reports on asylums and articles in medical journals follow a similar pattern of narrative strategies. All present a view of Indian psychiatry that is becoming modern and civilized, emerging out of the status of a ‘savage’ society.

## TEXTS FROM POSTCOLONIAL INDIA

The *Indian Journal of Neurology and Psychiatry* (later *Indian Journal of Psychiatry)* in its fourth year of publication carried a long article on the history of psychiatry in India and Pakistan.^28^ Varma was the first Assistant Superintendent of the Indian Mental Hospital at Kanke, Ranchi. He wrote a comprehensive chronological account of the changes brought in the Indian psychiatric institutions in Bengal, Bihar, Orissa, Madras, Calicut, Waltair, Mysore, Bombay, Sindh, Assam, Central Provinces, Punjab and Amritsar. Varma neither questioned the paradigms of western science nor did he look critically whether independent India would need more hospitals or any other alternative models.

In his presidential address at the Annual Conference of the Indian Psychiatric Society, Venkoba Rao deliberated on the ancient Indian thoughts on psychiatry.^29^ He attempted to relocate our psychiatric past from the pre-Vedic period to the post-Vedic Ayurvedic treatises, to show that it is in continuation with a uniform pattern all over the world. He tried his best to fit many indigenous categories with the western ones. But he never posed the problem to the practitioners of modern Indian psychiatry as to why and how we became alienated from such ancient learning. Two years later Rao^30^ would again talk about the sacred Hindu text *Gita* in relation to mental sciences and rediscover parallels with western psychiatry. He did not doubt that the *atma* and *kaya* duality of *Gita* can be different from a Descartian mind/body split, which is foundational to modern philosophy and cognitive science. He has also ignored Girindrasekhar Bose's significant contributions to Indian philosophy.^31^ In both the articles he has not questioned how these ancient sciences struggled with the western concept of mind and its disorders for its existence. What are its current forms? Rather, a seamless ancient past is invoked more as a nostalgic memory, whose remnants we cherish in the contemporary psychiatric discourse.

Somasundaram's article^32^ on the Indian Lunacy Act, 1912 is another example of a linear historical narrative that lamented why it took so long to change a colonial law, but failed to notice that Section 377 of Indian Penal Code which criminalizes homosexuality is not deleted. In a brief article, Michel Weiss^33^ pointed out the colonial hegemony over indigenous practices in the nineteenth century India. He observed that:

Competition between the Western and more popular indigenous medical practices was intense, and toward the end of the 19th century, the British asylum superintendents tended to look with increasing condescension and outright disdain at indigenous practices… This attitude…sanctioned the predominance of Western values in education and English over Indian languages and culture.^33^

Perhaps it was not before Chakraborty^34^ that a conceptual issue to judge our psychiatric knowledge through history and culture was raised. Brief in its scope, the article eruditely dealt with the complexity produced by discourses on colonialism, culture and psychiatry, and posed new theoretical questions.

## CONCLUSION

The *ahistorical* and *acultural* understanding has prevented us from exploring the conceptual issues that are specific to Indian psychiatry. We are still struggling to erase the *lack* in Indian psychiatry in relation to the Euro-American one.

Different perspectives of historicism have helped us to look at the epistemological struggles that took place at various levels of discourses of Indian psychiatry; Girindrasekhar Basu is one example. This critical investigation hints at the forgotten or silenced knowledge of Indian psychiatry that contributes to its conceptual development. If we need to know why the outcome of treatment of schizophrenia is better in our country compared to the developed ones, then we not only have to study the history and culture of schizophrenia in India, but also how schizophrenia is constructed in our society. Perhaps it is not too late to develop theoretical concepts of Indian psychiatry by exploring interdisciplinary historical studies visible at the horizon.

## Footnotes

* This is derived from the word *discourse* as used by Foucault. He seeks to account for the creation of objects within discourse 'by relating them to the body of rules that enable them to form as objects', and which thus constitute 'the conditions of their historical appearance'. This is significant in that he stresses the *constitutive* role of discursive *practices* in forming and determining objects, rather than the converse.^8^
